# Knowledge and Attitudes Toward Sports-Injury Prevention and Management Among Athletes and Coaches: A Cross-Sectional Study

**DOI:** 10.7759/cureus.96152

**Published:** 2025-11-05

**Authors:** Jaykumar Soni, Dixita Vora

**Affiliations:** 1 Musculoskeletal and Sports, College of Physiotherapy, Sumandeep Vidyapeeth Deemed to be University, Vadodara, IND

**Keywords:** athletes, athletic injuries, attitudes, first aid/education, health knowledge, knowledge and attitudes, practice, prevention of injury, sports injury prevention, cross-sectional studies

## Abstract

Introduction

Participation in sports benefits health and well-being, yet preventable injuries remain a common occurrence. We assessed the knowledge and attitudes of athletes and coaches regarding sports-injury prevention and management, and examined whether greater knowledge is associated with more favorable attitudes.

Method

We conducted a cross-sectional survey of athletes and coaches recruited by convenience sampling from sports complexes in Vadodara, India. Participants completed structured questionnaires assessing knowledge (scored against a predefined “satisfactory” threshold) and attitude (benchmarked as “positive”). We compared subgroup proportions for satisfactory knowledge and positive attitude using chi-square tests and evaluated the association between total knowledge and attitude scores using a two-tailed Pearson correlation, with statistical significance defined at conventional levels.

Results

Participants reported generally positive attitudes toward injury prevention; however, their knowledge was limited, particularly in areas such as basic first aid and fundamental management principles. Knowledge did not differ meaningfully across age, gender, practice frequency, injury history, or level of play. Coaches showed variable knowledge across domains. Among athletes, greater knowledge was associated with more favorable attitudes; this association was not evident among coaches.

Conclusion

Athletes and coaches endorsed prevention but demonstrated gaps in foundational knowledge. Linking education to practice appears promising, as greater knowledge among athletes is aligned with more favorable attitudes. Embedding targeted prevention content into coach education, providing structured athlete training in evidence-based first aid and safe training practices, and fostering team-wide safety culture may help translate intent into consistent preventive behavior. Future studies using broader sampling frames, validated instruments, and prospective injury surveillance can strengthen inference and guide implementation.

## Introduction

India has a long history of sport dating back to the Harappan civilization, where traditional games were integral to community recreation and physical fitness. Over time, the country has maintained a strong commitment to advancing sport nationally and internationally, with government initiatives aimed at nurturing talent and promoting performance excellence [1-5]. Within this ecosystem, athletes are the primary participants, and coaches are essential guides for skill development, goal achievement, and performance optimization (PhD Dissertation: Reed JP. Coach and Athlete Perceptions of an Athlete Monitoring and Strength and Conditioning Program; 2014) [6]. Participation in sport confers multiple health benefits, including lower risks of obesity, diabetes, and hypertension, as well as improved mental, emotional, and social well-being [7,8]. However, increased training intensity, particularly among young athletes, has led to a rise in sports-related injuries, which negatively affects individual performance and the overall growth of sports in India [9,10]. These injuries arise from intrinsic factors (e.g., age, gender, history of injury) and extrinsic factors (e.g., training conditions, equipment, and techniques) [11]. India reports a high incidence of sports injuries (58.9% to 73.4%), with male athletes being more commonly affected; and sprains, strains, and abrasions are frequent, particularly in the extremities such as the hands and ankles [12-14]. A major contributor to this issue is the limited awareness of injury prevention and management among athletes and coaches.

Effective prevention depends on structured training, appropriate supervision, and ongoing education, as knowledge directly shapes attitudes and practices [15-19]. Coaches play a pivotal role in fostering a culture of safety. Their understanding of injury mechanisms and appropriate responses can influence athlete behavior and reduce injury risk [20]. Yet, many coaching curricula do not sufficiently emphasize injury prevention, resulting in inconsistent implementation of safety strategies [21]. Incorporating targeted, sport-specific modules into coach education is essential for sustainable change [22]. Athletes also must engage in prevention by performing warm-ups, using the correct technique, and wearing appropriate protective gear. Strengthening knowledge and attitudes among both groups is crucial to reducing injury prevalence, enhancing performance, and promoting long-term participation. Therefore, this study aims to assess the knowledge and attitudes of athletes and coaches towards sports-injury prevention and management, compare these measures across key participant characteristics, and examine the association between knowledge and attitudes to inform targeted education and coaching interventions.

## Materials and methods

We conducted a cross-sectional study that recruited 150 participants (athletes and coaches) over a 12-month period from sports complexes in Vadodara city, Gujarat, India, using a convenience sampling approach. We calculated the sample size using the formula N=4pq/L², where p is the prevalence rate, q=1-p, and L is the allowable margin of error. The Institutional Review Board of Sumandeep Vidyapeeth Institutional Ethics Committee approved the study (approval no. SVIEC/ON/PHYS/BNMPT22/APRIL/23/15).

Eligibility criteria included athletes aged 18 to 40 years and coaches aged 18 to 60 years. We included male and female athletes actively engaged in sports at local, university, state, or national levels with at least six months of competitive experience, as well as coaches with at least one year of coaching experience. We excluded athletes with irregular participation and those involved in indoor games, which generally carry a lower risk of serious injury due to controlled environments, stable surfaces, and fewer unpredictable factors. In contrast, outdoor sports involve harsher conditions and longer exposures that are more likely to produce severe injuries.

After obtaining written informed consent and providing a participant information sheet, we collected data on demographics, history, frequency, and nature of sports-related injuries, as well as injury management strategies. Participants then completed structured questionnaires assessing their knowledge and attitudes toward sports-injury prevention and management, developed based on a previously published open-access instrument and adapted to suit the local context [12]. The knowledge questionnaire comprised 25 items; each correct response scored four points (maximum score of 100). A score of ≥60 indicated satisfactory knowledge. The instrument demonstrated strong content validity, with a Content Validity Index (CVI) >0.80. The attitude assessment included 18 items rated on a five-point Likert scale (total possible score of 90); higher scores indicated more favorable attitudes. The benchmark for a positive attitude was based on established studies, and the CVI for this tool also exceeded 0.80 [13].

We initially approached 150 individuals (140 athletes and 10 coaches). Three athletes declined participation due to time constraints and a reluctance to disclose their personal views, resulting in a final sample of 147 participants (137 athletes and 10 coaches).

We recorded data in Microsoft Excel (Microsoft Corp., Redmond, WA, US) and analyzed them using IBM SPSS Statistics for Windows, Version 28 (Released 2021; IBM Corp., Armonk, New York, United States). We summarized continuous variables as mean ± standard deviation (SD) and categorical variables as number (percentage). We used chi-square (χ²) tests to compare the proportion of participants with satisfactory knowledge (score ≥60) and with a positive attitude (per scale benchmark) across age, gender, practice frequency (days/week), sports-injury history, and level of play. To assess the association between total knowledge and attitude scores, we calculated two-tailed Pearson correlation coefficients (r). P-values tested the null hypothesis of zero correlation; statistical significance was defined as two-tailed P<0.05.

## Results

Participant characteristics 

A total of 147 participants took part in the study: 137 athletes and 10 coaches. Of these, 112 were male participants and 35 were female participants, and the mean age was 23 years. Overall, 87 participants reported a prior sports injury, and 60 reported no previous injury. Among coaches, seven were professional football coaches and three were professional hockey coaches; five trained national-level players, two trained local/state-level players, and one trained a district-level player. Male athletes experienced more sports injuries than female athletes (53.28%, n=73 vs. 10.28%, n=14, respectively).

Football and cricket showed the highest proportions of injured athletes among sports with meaningful sample sizes: football 17/19 (89.5%) and cricket 16/24 (66.7%), whereas hockey had 10/20 (50.0%) and “other” sports 39/67 (58.2%). Tennis registered 2/2 injuries (100%) and swimming 0/1 (0%), but these categories had very small denominators. The overall association between sport and injury did not reach statistical significance (χ²=11.134; P=.084; Table 1).

**Table 1:** Association between sports and injury in athletes ^*^P-values were calculated using Pearson χ² tests of independence (two-sided, α=.05); Fisher’s exact test was used when an expected cell count was <5. **Kabaddi, Khokho, Skating, and badminton.

Sports	Injury		Chi-Square	P-Value^*^
	Present	Absent		
Cricket	16	8	11.134	0.084
Football	17	2		
Hockey	10	10		
Swimming	0	1		
Tennis	2	0		
Volleyball	3	1		
Other**	39	28		

Injury was more common among athletes practicing five to six days per week (60/88; 68.2%) than among those practicing three to four days per week (17/29; 58.6%) or one to two days per week (4/8; 50.0%); and those training daily (seven days per week) had 6/12 injuries (50.0%). This gradient was descriptive only, the association between weekly practice frequency and injury was not statistically significant (χ²=2.703; P=.440; Table 2).

**Table 2:** Association between number of practice days and injury in athletes ^*^P-values were calculated using Pearson χ² tests of independence (two-sided, α=0.05); Fisher’s exact test was used when an expected cell count was <5.

Number of practice days/week	Injury		Chi-Square	P-Value^*^
	Present	Absent		
1-2 days	4	4	2.703	0.440
3-4 days	17	12		
5-6 days	60	28		
7 days	6	6		

Attitudes toward injury prevention were uniformly positive across the practice-frequency strata: 100% positive in the one-to-two and three-to-four days per week groups (8/8 and 29/29, respectively), 97.7% in the five-to-six days per week group (86/88), and 100% in the seven days per week group (12/12). The distribution of attitudes by practice frequency was not statistically significant (χ²=1.130; P=0.770; Table 3).

**Table 3:** Association between the number of practice days per week and the attitude ^*^P-values were calculated using Pearson χ² tests of independence (two-sided, α=0.05); Fisher’s exact test was used when an expected cell count was <5.

Number of practice days/week	Attitude		Chi-Square	P-Value^*^
	Positive attitude	Negative attitude		
1-2 days	8	0	1.130	0.770
3-4 days	29	0		
5-6 days	86	2		
7 days	12	0		

Knowledge of sports-injury prevention and management

Overall knowledge was low, with an average of 39.07% (n=57) of responses being correct and 61.5% (n=90) being incorrect. The highest proportion of correct answers (67.15%) pertained to bandaging a wound, whereas only 19.71% answered correctly on the principles of injury handling. Item-level knowledge performance for athletes and coaches is summarized in Table 4.

**Table 4:** Item-level knowledge on sports-injury prevention and management (athletes and coaches) Questionnaire adapted from Wang et al. (2012) [12].

Question	Answer	For athletes	For coaches
		N (%)	N (%)
1	Incorrect	95 (69%)	6 (60%)
	Correct	42 (30%)	4 (40%)
2	Incorrect	67 (48%)	3 (30%)
	Correct	70 (51%)	7 (70%)
3	Incorrect	64 (46%)	7 (70%)
	Correct	73 (53%)	3 (30%)
4	Incorrect	64 (46%)	4 (40%)
	Correct	73 (53%)	6 (60%)
5	Incorrect	99 (72%)	7 (70%)
	Correct	38 (27%)	3 (30%)
6	Incorrect	64 (46%)	7 (70%)
	Correct	73 (53%)	3 (30%)
7	Incorrect	110 (80%)	7 (70%)
	Correct	27 (19%)	3 (30%)
8	Incorrect	67 (48%)	9 (90%)
	Correct	70 (51%)	1 (10%)
9	Incorrect	77 (56%)	8 (80%)
	Correct	60 (43%)	2 (20%)
10	Incorrect	105 (76%)	6 (60%)
	Correct	32 (23%)	4 (40%)
11	Incorrect	98 (71%)	8 (80%)
	Correct	39 (28%)	2 (20%)
12	Incorrect	97 (70%)	7 (70%)
	Correct	40 (29%)	3 (30%)
13	Incorrect	93 (67%)	8 (80%)
	Correct	44 (32%)	2 (20%)
14	Incorrect	100 (72%)	8 (80%)
	Correct	37 (27%)	2 (20%)
15	Incorrect	53 (38%)	5 (50%)
	Correct	84 (61%)	5 (50%)
16	Incorrect	83 (60%)	6 (60%)
	Correct	54 (39%)	4 (40%)
17	Incorrect	63 (45%)	5 (50%)
	Correct	74 (54%)	5 (50%)
18	Incorrect	104 (75%)	9 (90%)
	Correct	33 (24%)	1 (10%)
19	Incorrect	93 (67%)	5 (50%)
	Correct	44 (32%)	5 (50%)
20	Incorrect	97 (70%)	8 (80%)
	Correct	40 (29%)	2 (20%)
21	Incorrect	45 (32%)	4 (40%)
	Correct	92 (67%)	6 (60%)
22	Incorrect	99 (72%)	7 (70%)
	Correct	38 (27%)	3 (30%)
23	Incorrect	68 (49%)	2 (20%)
	Correct	69 (50%)	8 (80%)
24	Incorrect	97 (70%)	5 (50%)
	Correct	40 (29%)	5 (50%)
25	Incorrect	82 (59%)	4 (40%)
	Correct	55 (40%)	6 (60%)

The knowledge of the coaches varied; for example, 70% correctly identified ligament damage, while only 10% answered other questions correctly.

Attitudes toward prevention and management

Participants reported generally positive attitudes toward injury prevention, with 43.78% strongly agreeing, 38.37% agreeing, 8.77% neutral, 5.7% disagreeing, and 3.51% strongly disagreeing. Among athletes, the strongest agreement (57.66%) was with the statement that stretching enhances flexibility and prevents injuries. The lowest agreement (29.93%) concerned whether injuries can be decreased through proper knowledge; this item also had the highest disagreement (7.3%). Full item distributions for athletes and coaches are presented in Table 5. Attitude did not differ significantly by type of sport (p=0.908), practice days (p=0.770), injury history (p=0.280), or player position (p=0.566).

**Table 5:** Item-level attitudes toward sports-injury prevention and management among both athletes and coaches Questionnaire adapted from Wang et al. (2012) [12].

Question	For athletes					For coaches				
	1	2	3	4	5	1	2	3	4	5
	N (%)	N (%)	N (%)	N (%)	N (%)	N (%)	N (%)	N (%)	N (%)	N (%)
1	3 (2.19%)	10 (7.30%)	13 (9.49%)	53 (38.69%)	58 (42.34%)	0 (0%)	1 (10%)	1 (10%)	5 (50%)	3 (30%)
2	1 (0.73%)	8 (5.84%)	14 (10.22%)	53 (38.69%)	61 (44.20%)	1 (10%)	1 (10%)	0 (0%)	5 (50%)	3 (30%)
3	1 (0.73%)	8 (5.84%)	8 (5.84%)	49 (35.77%)	71 (51.82%)	2 (20%)	0 (0%)	0 (0%)	4 (40%)	4 (40%)
4	6 (4.38%)	6 (4.38%)	10 (7.30%)	44 (32.17%)	71 (51.82%)	0 (0%)	0 (0%)	1 (10%)	6 (60%)	3 (30%)
5	3 (2.19%)	10 (7.30%)	14 (10.22%)	54 (39.42%)	56 (40.86%)	0 (0%)	2 (20%)	2 (20%)	3 (30%)	3 (30%)
6	2 (1.46%)	7 (5.11%)	12 (8.76%)	37 (27.01%)	79 (57.66%)	0 (0%)	1 (10%)	0 (0%)	4 (40%)	5 (50%)
7	9 (6.57%)	8 (5.84%)	9 (6.57%)	39 (28.47%)	72 (52.55%)	1 (10%)	0 (0%)	1 (10%)	4 (40%)	4 (40%)
8	9 (6.57%)	9 (6.57%)	9 (6.57%)	39 (28.47%)	71 (51.82%)	0 (0%)	1 (10%)	0 (0%)	5 (50%)	4 (40%)
9	6 (4.38%)	9 (6.57%)	15 (10.95%)	54 (39.42%)	57 (41.61%)	0 (0%)	1 (10%)	1 (10%)	6 (60%)	2 (20%)
10	4 (2.92%)	9 (6.57%)	13 (9.49%)	59 (43.07%)	52 (37.96%)	0 (0%)	1 (10%)	0 (0%)	6 (60%)	3 (30%)
11	4 (2.92%)	8 (5.84%)	10 (7.30%)	65 (47.45%)	50 (36.50%)	1 (10%)	1 (10%)	0 (0%)	6 (60%)	2 (20%)
12	8 (5.84%)	3 (2.19%)	7 (5.11%)	56 (40.88%)	63 (45.99%)	1 (10%)	0 (0%)	0 (0%)	6 (60%)	3 (30%)
13	8 (5.84%)	5 (3.65%)	11 (8.03%)	56 (40.88%)	57 (41.61%)	0 (0%)	0 (0%)	1 (10%)	5 (50%)	4 (40%)
14	5 (3.65%)	10 (7.30%)	11 (8.03%)	61 (44.53%)	50 (36.50%)	0 (0%)	0 (0%)	2 (20%)	7 (70%)	1 (10%)
15	3 (2.19%)	7 (5.11%)	18 (13.14%)	59 (43.07%)	50 (36.50%)	0 (0%)	0 (0%)	4 (40%)	3 (30%)	3 (30%)
16	1 (0.73%)	4 (2.92%)	10 (7.30%)	53 (38.69%)	69 (50.36%)	0 (0%)	0 (0%)	1 (10%)	6 (60%)	3 (30%)
17	10 (7.30%)	15 (10.95%)	18 (13.14%)	53 (38.69%)	41 (29.93%)	0 (0%)	0 (0%)	1 (10%)	4 (40%)	5 (50%)
18	4 (2.92%)	5 (3.65%)	14 (10.22%)	62 (45.26%)	52 (37.96%)	0 (0%)	1 (10%)	1 (10%)	4 (40%)	4 (40%)

Coaches also exhibited generally positive attitudes, particularly toward stretching and knowledge-based prevention: 50% strongly agreed with these points, whereas only 10% strongly agreed regarding responsibilities related to cardiopulmonary resuscitation. The highest negative response (20%) concerned maintaining physical and mental well-being. Attitude scores ranged from 41 to 90.

Associations and correlations

Knowledge did not differ significantly by years of play (p=0.426), practice days per week (p=0.481), injury history (p=0.117), or player level (p=0.337). Knowledge correlated positively with attitude among athletes (r=0.232; p=0.006) but not among coaches (r=0.5; p=0.141), as shown in Table 6.

**Table 6:** Correlation between knowledge and attitudes in athletes and coaches P-values calculated using two-tailed Pearson correlation between the total knowledge and attitude scores.

Relation between knowledge and attitude questionnaire	R-Value	P-Value	Result
Athletes	0.232	0.006	Weak Positive
Coaches	0.5	0.141	Not Significant

Consistent with this pattern, on the attitude item regarding safety devices, 51.82% agreed and 4.38% disagreed, aligning with a related knowledge item for which 30.66% answered correctly. Although 69.3% of athletes reported receiving advice on protective aids from coaches, coaches themselves often lacked a comprehensive understanding of prevention strategies. Knowledge scores in this section ranged from four to 80, indicating substantial variability.

## Discussion

This study evaluated the knowledge and attitude of athletes and coaches regarding sports injury prevention and management to inform strategies for reducing injury incidence and improving performance. We enrolled 147 participants (137 athletes and 10 coaches) and used a structured questionnaire to capture demographic characteristics, sports involvement, and both knowledge and attitude measures.

Demographic profile and injury patterns

Male participants experienced a higher rate of sports injuries than female participants (53.28%, n=73 vs. 10.28%, n=14), consistent with earlier reports that attribute male predominance to longer practice hours and greater exposure [3,8,9]. Prior literature has linked injuries to factors such as suboptimal attitudes, inadequate warm-up routines, and improper use of equipment. Sreekaarini et al. noted overtraining and insufficient warm-up as major contributors among student athletes [9]. In our cohort, football and cricket had the highest injury rates, whereas tennis and swimming had the lowest, mirroring findings by Gogoi et al. [23] and Kerr et al. [24]. Regarding training frequency, 25.18% (n=37) practiced ≤4 days/week, 59.86% (n=88) >5 days/week, and 8.16% (n=12) daily. Among injured participants, 62.86% (n=86) practiced frequently, suggesting a possible relationship between higher practice frequency and injury prevalence. Wang et al. reported that more days of practice improved task performance via increasing awareness and motor-sensory skills and reducing injuries [12]; however, our results did not show this association.

Knowledge of sports-injury prevention and management

Knowledge levels were low, with an average correctness of only 39.07% (n=57) of responses, ranging from the highest correctness for wound bandaging (67.15%) to the lowest for principles of injury handling (19.71%). These findings indicate gaps in basic first aid and injury management knowledge. Bakar et al. similarly reported limited knowledge of first aid among athletes, particularly for fractures [11]. We observed no significant gender differences in knowledge of sports-injury prevention and management, which contrasts with Wang et al., who found female athletes had better knowledge than male athletes [12]. No statistically significant associations were detected between years of play and knowledge (p=0.426), practice days per week and knowledge (p=0.481), injury history and knowledge (p=0.117), or player level and knowledge (p=0.337). These results differ from those of Sohail et al., who linked longer sports involvement to improved knowledge [13], and from Shamlaye et al., who emphasized the influence of peers and coaches on injury awareness [14].

Coaches’ knowledge also varied; for example, 70% correctly identified ligament damage, while only 10% answered other items correctly. Zech et al. suggested that a lack of formal training may contribute to this gap [17].

Attitude toward injury prevention and management

Despite limited knowledge, attitudes were generally positive. Among athletes, the strongest agreement was that stretching enhances flexibility and prevents injuries, whereas the lowest agreement concerned whether injuries can be decreased through proper knowledge. Attitude was not significantly associated with the type of sport, the number of practice days, injury history, or player position. These findings align with those of McKay et al., who found no association between beliefs and demographics, such as age or competitive level [25]. However, Short et al. reported that injury history influenced perceived risk in collegiate female athletes [26], Finch et al. underscored the value of protective equipment [16], and Anyadike-Danes et al. emphasized the roles of load management and coach education in shaping attitudes [27].

Correlation between knowledge and attitude

Knowledge correlated significantly with attitude among athletes (p=0.006) but not among coaches (p=0.141). Participants with better knowledge tended to hold more favorable attitudes toward injury management. For instance, 51.82% agreed and 4.38% disagreed with the attitude item on safety devices, which aligned with a related knowledge item answered correctly by 30.66%. Pedlar et al. observed that injury history can shape both knowledge and attitude [28]. Understanding, practice, and prior experience with injury care influence behavior and perceptions of prevention [20-22]. Szabó et al. reported that negative attitudes increased the risk of injury [19] while Steffen et al. found that footballers had good knowledge but a poor attitude [29]. Proper warm-ups were widely endorsed yet poorly understood, highlighting a persistent knowledge-practice gap. McKay et al. further emphasized that positive attitudes correlate with fewer injuries, consistent with the present findings [25].

Limitations

Our study has several important limitations worth considering. First, the cross-sectional design precludes causal inference; observed correlations between knowledge and attitude cannot establish directionality. Second, we used convenience sampling from sports complexes in a single city, excluding indoor sports; as a result, the findings may not be generalizable to other regions, levels of play, or sport types. Third, the coach subgroup was small and unevenly distributed by sport, limiting power for subgroup analyses and the stability of those estimates. Fourth, all measures relied on self-report, including prior injury history, which introduces recall and social-desirability bias; injuries were not verified against medical records, and we did not capture injury severity or time at risk (exposure hours). Fifth, although the questionnaires demonstrated acceptable content validity, we did not comprehensively assess psychometric properties in this sample (e.g., internal consistency, test-retest reliability, and construct validity). Sixth, dichotomizing continuous scores to define “satisfactory knowledge” and “positive attitude” may have introduced information loss and misclassification, and the chosen cut points may not reflect clinically meaningful thresholds. Seventh, group comparisons were primarily conducted using univariable (chi-square tests) and bivariate (correlations) analyses, without multivariable adjustment for potential confounders such as training load, prior injuries, sport specialization, or access to medical care. Eighth, multiple comparisons were conducted without formal adjustment, which may increase the risk of type I error. Finally, correlation testing assumes that the data follow appropriate distributional conditions; if normality is not met, nonparametric methods would be more suitable. Future studies using probability sampling across diverse settings, prospective injury surveillance with standardized definitions and exposure metrics, fully validated instruments, and adjusted multivariable models would strengthen inference and generalizability.

## Conclusions

This study evaluated the knowledge and attitudes of athletes and coaches regarding sports injury prevention and management to determine whether greater knowledge is associated with more favorable attitudes. We found that participants generally held positive attitudes toward prevention; however, their knowledge was limited, particularly in areas such as first aid and fundamental management principles. Among athletes, higher knowledge was associated with more favorable attitudes, suggesting that education can shape beliefs and intended behaviors. Coaches showed variable knowledge, underscoring gaps in training that may affect how safety practices are taught and reinforced. These findings have practical relevance for sports medicine and public health. Embedding targeted injury-prevention content into coach education, delivering structured athlete education on evidence-based first aid and safe training practices, and promoting a safety culture across teams and facilities can support safer participation and sustained performance. Future work should standardize training modules, evaluate their effectiveness over time, and explore how multidisciplinary collaboration among clinicians, coaches, and sport organizations can translate knowledge into consistent preventive practice.
